# High-frequency taVNS modulates olfaction-related brain activity in patients with subjective cognitive decline

**DOI:** 10.3389/fnhum.2026.1888005

**Published:** 2026-08-03

**Authors:** Jiazhu Huang, Xiaocheng Li, Kaixuan Zhou, Bo Liu, Yichen Wei, Huanting Guo, Shanshan Wei, Shaojie Xu, Ying Liu, Jinli Huang, Demao Deng, Lingyan Liang

**Affiliations:** 1 Department of Radiology, The People's Hospital of Guangxi Zhuang Autonomous Region, Guangxi Academy of Medical Science, Nanning, Guangxi, China; 2 Department of Neurology, The People’s Hospital of Guangxi Zhuang Autonomous Region, Guangxi Academy of Medical Sciences, Nanning, Guangxi, China

**Keywords:** functional connectivity, orbitofrontal cortex, regional homogeneity, subjective cognitive decline, transcutaneous auricular vagus nerve stimulation

## Abstract

**Background:**

Subjective cognitive decline (SCD) is considered a high-risk factor for Alzheimer’s disease and serves as a critical window of the prevention and treatment. Transcutaneous auricular vagus nerve stimulation (taVNS) has shown therapeutic effects on cognitive impairment, but there is a lack of research on its application in SCD.

**Purpose:**

This study aimed to explore the immediate modulatory effects of taVNS at different frequencies on the brain function in patients with SCD.

**Methodology:**

Seventy SCD and 49 healthy control (HC) were enrolled. Resting-state functional MRI data were collected at baseline and during taVNS with three stimulation conditions: 1 Hz-taVNS, 20 Hz-taVNS, and sham taVNS (staVNS), respectively. Regional homogeneity (ReHo) analysis was used to identify spontaneous neural activity changes in SCD; the abnormal brain regions were then used as seed for subsequent functional connectivity (FC) analysis. In addition, ReHo and FC analyses were performed to assess the immediate modulatory effects of taVNS in SCD.

**Results:**

Compared with HC, SCD showed abnormal ReHo and FC mainly involving olfaction-related brain regions, and the reduced value was positively associated with Shape Trail Test-part B. During taVNS, we found that 20 Hz-taVNS significantly increased ReHo in the bilateral olfactory cortex compared with baseline, and decreased FC between the left medial orbitofrontal cortex and the right precentral and postcentral gyri. No significant ReHo or FC changes were observed during 1 Hz-taVNS and staVNS.

**Conclusion:**

These findings suggest that SCD exhibits reduced spontaneous activity and disrupted FC in the olfaction-related brain regions, which may reflect early functional alterations before objective impairment; 20 Hz-taVNS can modulate these cerebral functional abnormalities and may hold potential as a noninvasive neuromodulation strategy in SCD.

## 1 Introduction

According to the World health statistics 2024, Alzheimer’s disease (AD) and other dementia rose to the 6th leading cause of death, ranking 4th among high-income populations in the European Region and the Western Pacific Region (World Health Organization, 2024). Current anti-Aβ treatments are restricted to select early AD cohorts with suboptimal efficacy, and biomarker validation is a mandatory prerequisite for administration. Furthermore, irreversible neural damage may have already occurred in mild cognitive impairment (MCI), a state between normal aging and dementia (Jessen et al., 2014). Accordingly, subjective cognitive decline (SCD), lying in the early disease spectrum, is identified as the key window for targeted prevention and clinical intervention. SCD is characterized by self-perceived deterioration in cognitive functions with a normal range in objective neuropsychological assessments, without any acute event (Jessen et al., 2014). It serves as a high-risk factor for AD and may correspond to state 2 of AD according to the recently revised criteria (Jack et al., 2024).

Transcutaneous auricular vagus nerve stimulation (taVNS) is based on classical VNS and aligns with traditional Chinese auricular acupuncture. TaVNS delivers pulsed electrical stimulation to the auricular branch of the vagus nerve, leading to neurotransmitter release and neuroplasticity in brain networks. Study has shown that stimulation at the cymba conchae effectively activates the vagal pathway (Yakunina et al., 2017), especially the nucleus of solitary tract and locus coeruleus (LC), resulting in the improvement of cognitive and emotional functions. Aidan J. Murphy and colleagues found that taVNS in patients with MCI modulated the functional connectivity (FC) within the semantic network, salience network, hippocampus, and other cortical and subcortical regions (Murphy et al., 2023). A recently double-blind randomized clinical trial revealed that taVNS significantly improved cognitive performance of MCI (Wang et al., 2022). These findings indicate the great potential of taVNS in cognitive decline. Furthermore, taVNS offers advantages including non-invasive nature, low cost, ease of use, and favorable safety and tolerability profile, promoting its clinical application (Kim et al., 2022). However, clinical studies on taVNS in SCD remain scarce.

Functional MRI (fMRI) enables non-invasive detection of neural activity *in vivo* and has been widely used in the study of human advanced cognitive function. It is recognized as the most effective method for evaluating the central response to VNS (Broncel et al., 2020). Regional homogeneity (ReHo) is highly sensitive to the synchronization of neural activity within local brain regions, requiring no *a priori* definition of region of interest and possessing excellent test–retest reliability (Yang et al., 2020). FC serves as an effective approach to uncover the organizational characteristics of the human brain (Buckner et al., 2013). Although previous neuroimaging reviews have suggested that brain function abnormalities in SCD are often located in the default mode network, there remains substantial heterogeneity across studies, and no consistent imaging biomarkers have yet been identified for SCD diagnosis (Wang et al., 2020).

The present study employed ReHo and FC analyses to investigate the resting-state brain functional characteristics of SCD, thereby providing neuroimaging evidence for early diagnosis. Furthermore, we explored the immediate effect of high- and low-frequency taVNS in patients with SCD. We hypothesis that SCD is associated with abnormal function of cognition-related brain regions, and that these abnormalities would be modulated by taVNS, particularly during high-frequency stimulation.

## 2 Materials and methods

This study was approved by the Ethics Committee of the People’s Hospital of Guangxi Zhuang Autonomous Region (KY-GZR-2021-104). It has been registered in the Chinese Clinical Trial Registry (ChiCTR2200058427). Written informed consent was acquired from all participants, and all procedures were performed in accordance with the Declaration of Helsinki.

### 2.1 Participants

A total of 70 individuals with SCD and 49 healthy control (HC) were enrolled, with age, sex and years of education matched. Inclusion criteria were as follow: (1) Age 55–75 years old; (2) Right-handedness; (3) Normal performance on the standard neuropsychological tests (used to screen for MCI and prodromal AD); (4) No hearing, visual, or language communication impairment that would interfere with the completion of study assessments.

The exclusion criteria included: (1) Diagnosis of MCI or AD; (2) Central nervous system conditions, such as brain tumors, Parkinson’s disease, encephalitis, epilepsy, etc.; (3) Other systemic diseases that may cause cognitive impairment, such as thyroid dysfunction, severe anemia, hepatic encephalopathy, syphilis, HIV, etc.; (4) Psychiatric disorders (e.g., depression, anxiety) or substance abuse; (5) Participates who have used any drugs potentially affecting cognitive function or inducing failure of vital organs (heart, brain, kidney, etc.) within the specified washout period; (6) Relevant cardiovascular and cerebrovascular risk factors, such as a history of hypertension, dyslipidemia, diabetes mellitus, etc. (7) MRI contraindications, presence of non-removable metallic dental restorations or other metallic implants, claustrophobia, etc. (1) Participants would be classified as SCD (2) if they have self-reported memory decline within the last 5 years or reported and confirmed by a reliable informant, show a feeling of worse cognitive performance than others of the same age group and express concern about it; otherwise, they would be assigned to HCs group. The neuropsychological tests including the Subjective Cognitive Decline Questionnaire (SCD-Q), Chinese version of Mini-Mental State Examination (MMSE), Montreal Cognitive Assessment-Basic (MoCA-B) Chinese version, Auditory Verbal Learning Test (AVLT), Shape Trail Test (STT), Animal Fluency Test (AFT), Boston Naming Test (BNT).

### 2.2 MRI protocols

The MRI data were acquired using a 3.0 T Siemens MAGNETOM Vida scanner (Siemens Healthineers). To avoid head movement, each participant’s head was immobilized by foam pads in a standard 64-channel birdcage head coil. Resting state fMRI (rs-fMRI) images were obtained with EPI sequence with the following parameters: repetition time (TR)/echo time (TE) = 2,000 ms/30 ms, field of view (FOV) = 220 mm × 220 mm, flip angle = 90°, matrix size = 88 × 88, slice thickness 2.5 mm, 66 slices. T1-weighted images: 3D MPRAGE sequence, TR/TE = 2,000 ms/2.04 ms, FOV = 256 mm × 256 mm, flip angle = 7°, matrix size = 256 × 256, slice thickness 1 mm, and 196 slices.

### 2.3 Concurrent taVNS/fMRI system

We employed a participant-blinded, sham-controlled crossover design for concurrent taVNS/fMRI protocol. Concurrent taVNS and rs-fMRI were conducted for SCD patients. Each SCD patient then received all three taVNS conditions [20 Hz-taVNS, 1 Hz-taVNS and sham taVNS (staVNS)] in a randomized order at an interval of 24 h within 1 week. The taVNS stimulator (TENS-200) was used with the following parameters: (1) Stimulation frequencies of 20 Hz and 1 Hz, with a pulse width of 250 μs; (2) Current intensity ≤ 3.0 mA, determined by individual tolerance of the patients; (3) Stimulation duration of 8 min. The electrode was place on the auricular concha (distribution of afferent branch of the VN) of the left ear for the 20 Hz-/1 Hz-taVNS, while on the left superior scapha (free of vagus innervation) for staVNS.

### 2.4 Image data processing

Preprocessing of rs-fMRI data were performed using Data Processing & Analysis for Brain Imaging (DPABI, version 6.0) implemented in MATLAB. The first 10 volumes were discarded, and the remaining images were subjected to slice-timing correction and realignment to the first volume. Participants with head motion exceeding 3 mm translation or 3° rotation in any direction were excluded. Functional images were normalized to MNI space using T1-weighted image-based segmentation, and resampled to 3 mm × 3 mm × 3 mm voxels to ensure spatial standardization. Nuisance signals, including motion parameters and average signals from white matter and ventricles, were regressed out to reduce non-neuronal noise. Additionally, linear trends were removed, and temporal band-pass filtering (0.01–0.08 Hz) was applied.

ReHo Analysis: ReHo maps were computed using the DPABI toolbox by calculating Kendall’s coefficient of concordance (KCC) between each voxel and its neighboring voxels. ReHo values were standardized by subtracting the global mean ReHo value and dividing by the global standard deviation. Subsequently, the resulting KCC-ReHo values were converted to *z*-scores to facilitate statistical analysis, and spatially smoothed using a 6 mm full-width at half-maximum (FWHM) Gaussian kernel to reduce spatial noise and enhance the signal-to-noise ratio of the ReHo maps.

FC Analysis: Regions with altered ReHo were defined as regions of interests (ROIs). The mean blood oxygen level-dependent (BOLD) time series from each region was extracted and correlated with the BOLD time series of all other brain voxels using Pearson correlation. The obtained correlation coefficients were converted to *z*-scores via Fisher’s *r*-to-*z* transformation to improve the normality of the data distribution, thereby obtaining whole-brain FC maps. Finally, a 6 mm FWHM Gaussian kernel was used for spatial smoothing to reduce spatial noise and enhance the reliability of FC results.

### 2.5 Statistical analysis

Demographic and clinical data were compared between the SCD and HC groups using the Mann–Whitney *U* test or the chi-squared test with SPSS software (version 25, SPSS Inc., Armonk, NY, USA). Categorical variables (e.g., sex) were compared via chi-square tests. The Shapiro–Wilk test was used to assess the normality of data distribution. Nonparametric Mann–Whitney *U* tests were applied to non-normally distributed variables, including age, years of education, MMSE, MoCA-B, SCD-Q, AVLT, BNT, STT-A/B.

Differences in ReHo and FC values between the SCD and HC groups were evaluated using independent-samples *t*-tests in DPABI running on the MATLAB platform. For these imaging results, Gaussian random field (GRF) correction was applied for multiple comparisons, with a voxel-level *p* < 0.001 and a cluster-level *p* < 0.05.

FC was calculated for all three stimulation conditions (1 Hz-taVNS, 20 Hz-taVNS, and sham taVNS [staVNS]) using ROIs based on the brain regions showing altered ReHo between the SCD and HC groups. ReHo values were also calculated under the same three stimulation conditions. To assess the effects of different stimulation conditions on brain activity in the SCD group, a two-way repeated-measures analysis of variance (ANOVA) was conducted using a flexible factorial design in SPM12. False Discovery Rate (FDR) correction was applied at *p* < 0.05 to identify significant effects of stimulation conditions. For stimulation conditions showing a significant *F*-test result, *post hoc* paired *t*-tests were performed to compare differences among the 1 Hz-taVNS, 20 Hz-taVNS, and staVNS conditions. GRF correction was also applied to these post hoc results for multiple comparisons (voxel-level *p* < 0.001, cluster-level *p* < 0.05).

For each participant, clusters showing significant group differences in ReHo and FC between the SCD and HC groups were selected as ROIs. The normalized ReHo and FC values of these ROIs were extracted for correlation analysis. ROI-based correlation analyses between these normalized values and neuropsychological test scores (which exhibited significant group differences) were conducted separately for SCD patients and HCs using Spearman correlation analysis in SPSS, with statistical significance set at *p* < 0.05.

## 3 Results

### 3.1 Demographic and clinical characteristics

Four SCD participants and three HCs were excluded due to severe head motion artifacts. Ultimately, 66 SCD patients and 46 healthy controls were enrolled. Age, sex and education level were comparable between groups (*p* > 0.05). All participants had normal cognitive functions. The SCD group obtained significantly higher SCD-Q scores (*p* < 0.001), with full data listed in Table 1.

**Table 1:** Demographic characteristics and neuropsychological assessment results.

	SCD (*n* = 66)	HC (*n* = 46)	*Z/x* ^2^	*p*
Age (years)	60 (58, 64)	60 (57, 66)	−0.157	0.875
Sex (M/F)	16/50	18/28	2.842	0.092
Education (years)	12.0 (7.0, 15.0)	12.0 (9.0, 15.0)	−1.408	0.159
Neuropsychological tests				
SCD-Q	5.0 (3.5, 5.5)	1.0 (0.0, 2.1)	−7.831	<0.001
MMSE	29.0 (28.0, 30.0)	29.0 (28.0, 30.0)	−0.655	0.512
MoCA-B	25.0 (23.0, 27.0)	25.0 (23.0, 26.0)	−0.042	0.967
AVLT-N5	5.0 (4.0, 6.0)	5.0 (5.0, 7.0)	−1.719	0.086
AVLT-N7	21.0 (19.0, 22.0)	22.0 (20.0, 23.0)	−1.450	0.147
STT-A	64.0 (50.0, 83.3)	63.0 (51.5, 91.2)	−0.163	0.871
STT-B	146.5 (122.8, 198.5)	147.0 (112.5, 180.0)	−0.819	0.413
AFT	16.0 (13.0, 18.0)	16.0 (14.0, 19.0)	−0.801	0.423
BNT	25.0 (22.0, 28.0)	25.0 (22.8, 28.0)	−0.098	0.922

SCD, subjective cognitive decline; HC, healthy control; SCD-Q, subjective cognitive decline questionnaire; MMSE, Mini-Mental State Examination; MoCA-B, Montreal Cognitive Assessment-Basic; AVLT-N5, Auditory Verbal Learning Test: 20 min long-term delayed recall; AVLT-N7, Auditory Verbal Learning Test: recognition; STT, Shape Trail Test; AFT, Animal Fluency Test; BNT, Boston Naming Test.

### 3.2 Regional brain activity and FC characteristics in SCD at baseline

**Figure 1:**
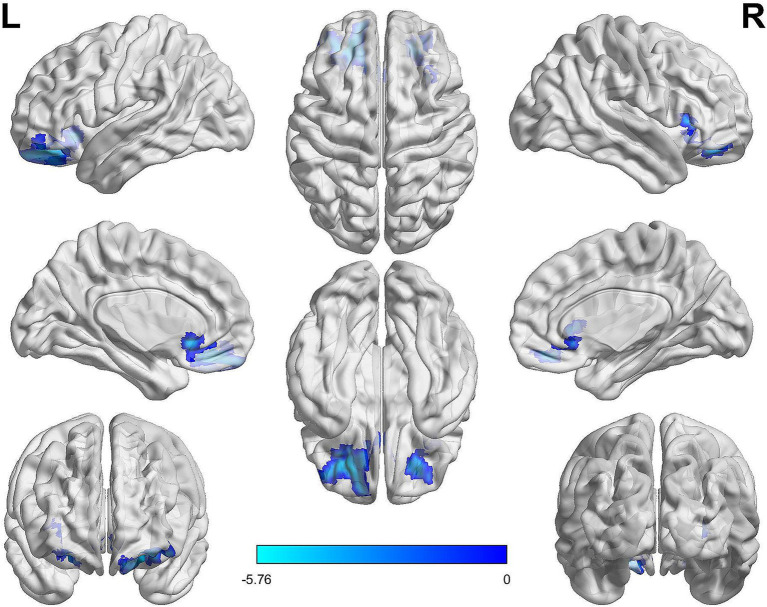
Changes of regional brain activity in SCD. Reduced ReHo was primarily observed across extensive regions of the prefrontal cortex.

Compared with HC, patients with SCD showed significantly reduced ReHo values in multiple brain regions, including the bilateral anterior orbital gyrus, bilateral medial orbital gyrus, left middle frontal gyrus, left caudate nucleus, left olfactory cortex, left superior frontal gyrus, the left posterior and lateral orbital gyrus, as well as the right insula (*p* < 0.05, GRF correction, Cohens_d 1.11), as illustrated in Figure 1.

**Figure 2:**
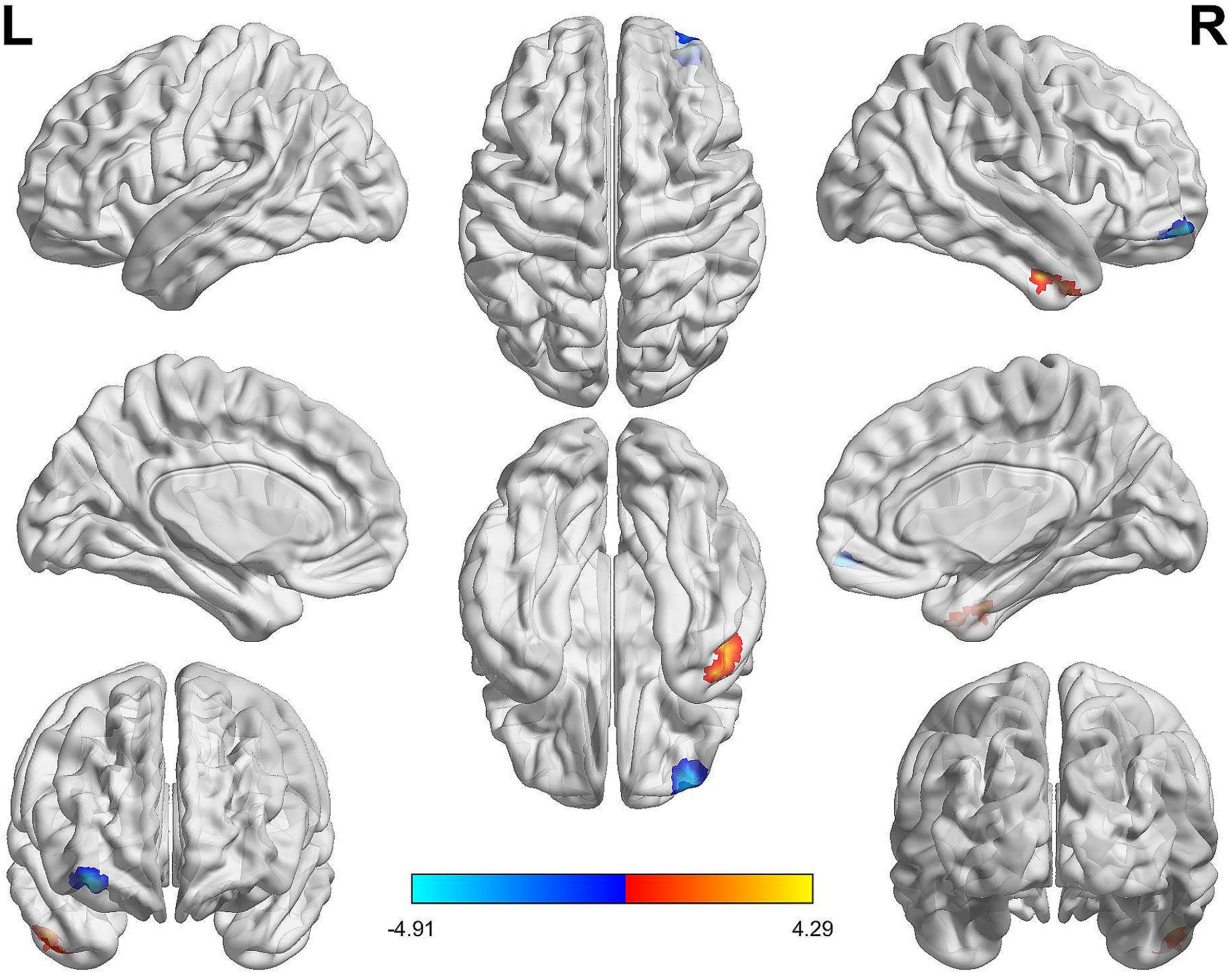
FC of the left ORBmid changed in SCD, it decreased in the anterior orbitofrontal cortex, while increased in the right inferior temporal gyrus. ORBmid: Orbital part of the middle frontal gyrus.

Given that most abnormal voxels were localized in the orbital part of the middle frontal gyrus (ORBmid) based on the AAL-90 brain atlas, we selected the left and right ORBmid as the seed region for subsequent FC analysis. Relative to HC, the SCD group exhibited reduced FC between the left ORBmid and the right anterior orbital cortex and enhanced FC between the left ORBmid and the right inferior temporal gyrus (all *p* < 0.05, GRF corrected, Cohens_d 0.95 and 0.83, respectively). These results are displayed in Figure 2. While there was no significant changes of the FC of the right ORBmid.

**Figure 3:**
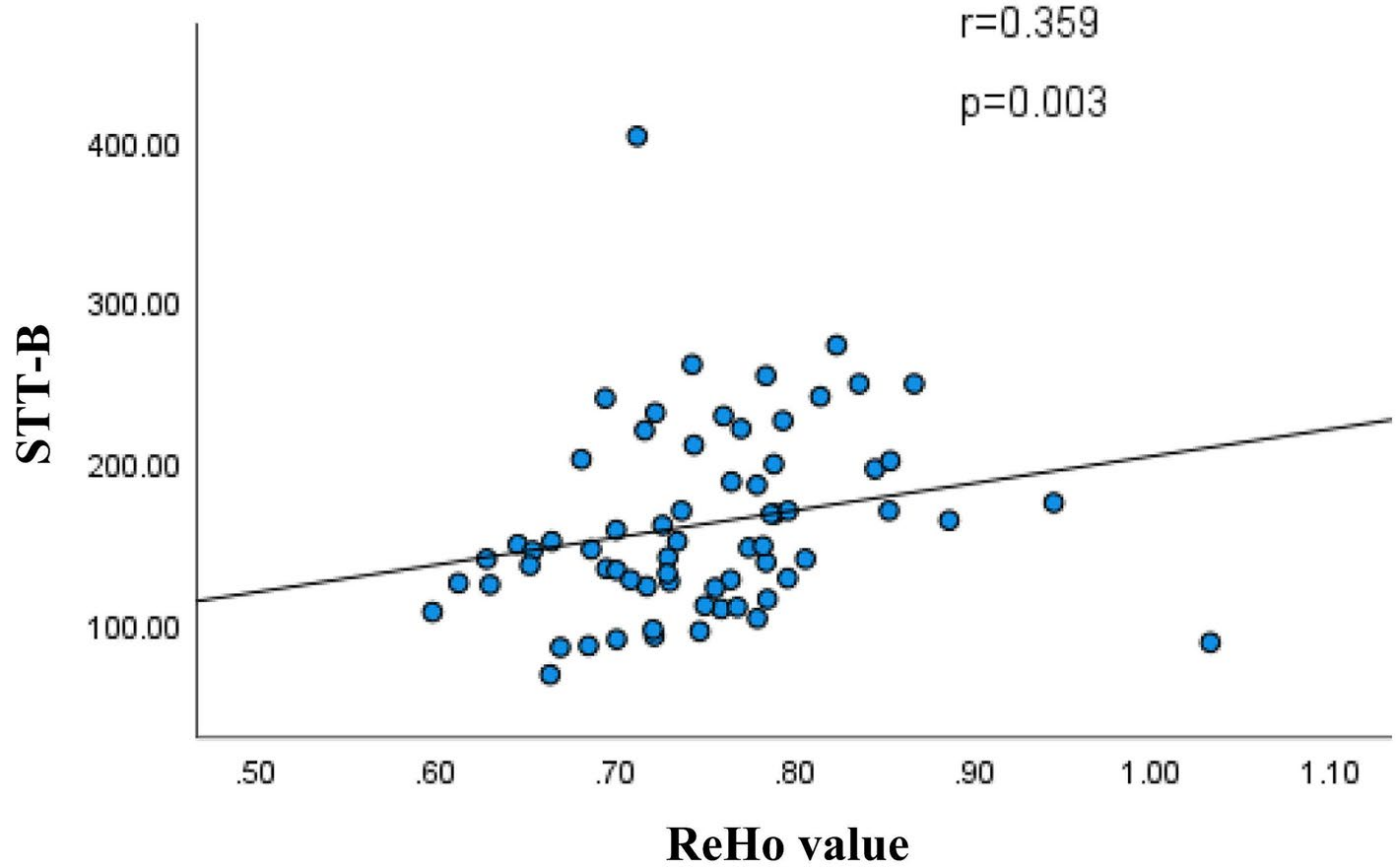
Correlation analysis between ReHo values of the abnormal brain regions and neuropsychological test scores. There is a positive correlation between ReHo values of the abnormal brain regions and STT-B score. STT-B, Shape Trail Test-part B.

ReHo values in regions showing abnormal spontaneous activity were positively associated with STT_B scores (*r* = 0.359, *p* = 0.003). No significant correlations were observed between altered FC values and neuropsychological test indices (Figure 3).

### 3.3 Immediate effects of taVNS on brain function in SCD patients

**Figure 4:**
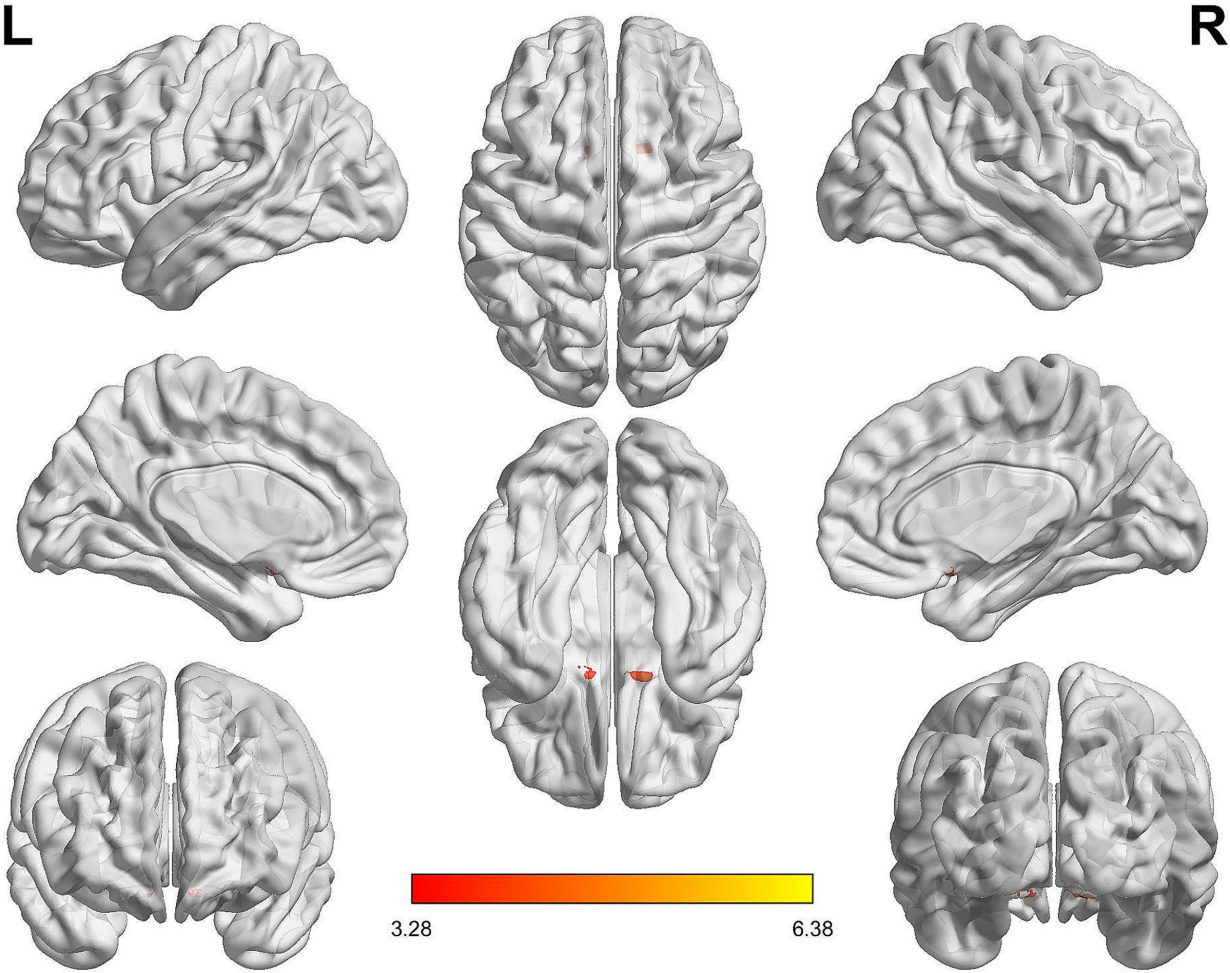
Modulation of regional brain activity in SCD patients by 20 Hz-taVNS. Compared with baseline, regional brain activity in the bilateral olfactory cortices was significantly enhanced during 20 Hz-taVNS.

Compared with baseline, 20 Hz-taVNS significantly increased ReHo value in the bilateral olfactory cortex [*p* < 0.05, GRF corrected, Cohens_d 0.75 (L) and 1.13 (R)], whereas 1 Hz-taVNS and sham taVNS failed to induce significant changes in brain activity (Figure 4).

**Figure 5:**
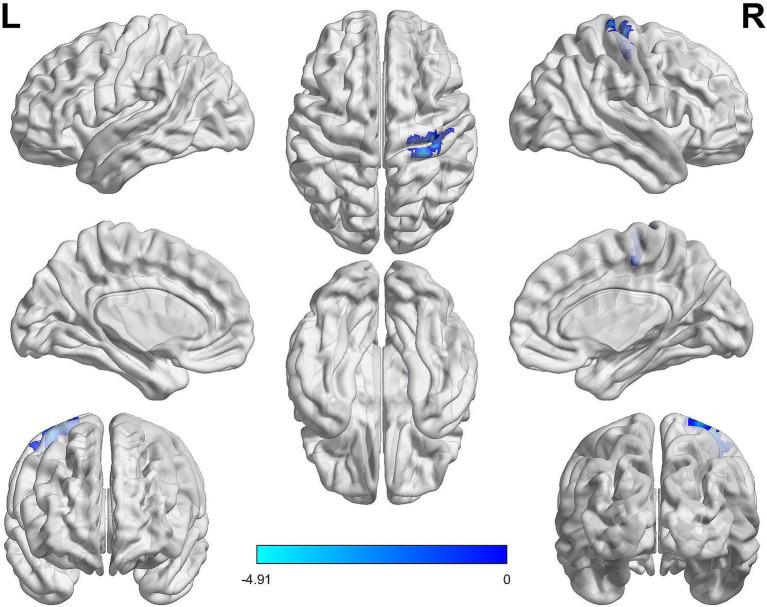
Modulation of the FC of the left ORBmid by 20 Hz-taVNS in SCD. 20 Hz-taVNS reduced FC between the left ORBmid and the right precentral and postcentral gyri compared with baseline. ORBmid: Orbital part of the middle frontal gyrus.

Using the left ORBmid as seed region for FC analysis, we found that 20 Hz-taVNS induced decreased FC with the right precentral gyrus and postcentral gyrus (*p* < 0.05, GRF corrected, Cohens_d 0.77). No significant changes in regional brain activity or FC were observed during 1 Hz-taVNS or staVNS. These results are presented in Figure 5.

## 4 Discussion

This study employed rs-fMRI with ReHo and FC analysis to investigate brain functional characteristics of SCD and the immediate modulatory effects of taVNS in SCD patients. The results showed that SCD patients exhibited decreased regional spontaneous brain activity primarily involving the prefrontal cortex, which is associated with cognitive function. FC of the left ORBmid was decreased in the right anterior orbital cortex and increased in the right inferior temporal gyrus. During 20 Hz-taVNS, local brain activity in bilateral olfactory cortex was enhanced compared with baseline, whereas FC between the left ORBmid and the right precentral and postcentral gyri decreased. No significant changes in local brain activity or FC were observed during 1 Hz-taVNS or staVNS.

The most significantly abnormal brain regions in SCD patients are overlapped in orbital frontal cortex (OFC), including bilateral anterior orbital gyrus, bilateral medial orbital gyrus, as well as the left posterior and lateral orbital gyrus. Remarkably, the OFC is a critical secondary olfactory region (Pacyna et al., 2023), involved in central olfactory processing (Jobin et al., 2023). Olfactory dysfunction closely correlates with cognitive impairment; objective olfactory deficits are more prevalent than patients’ subjective complaints (Lian et al., 2019; Devanand et al., 2010). AD patients exhibit olfactory deficits before cognitive symptoms arise (Dan et al., 2021). Longitudinal studies have shown that faster olfactory decline in cognitively normal elderly predicts higher rates of progression to MCI and dementia, smaller olfaction-related brain regions including OFC, and reduced gray matter volume in AD (Pacyna et al., 2023). Rs-fMRI studies show decreased ReHo in right OFC in AD patients, with donepezil treatment partially restoring activity but remaining lower than healthy controls (Dai et al., 2024). MCI patients also exhibit atrophy in olfactory processing regions, notably limbic and medial temporal lobes, which may predict early cognitive decline in AD (Jobin et al., 2023). Olfactory recognition and discrimination tests can differentiate MCI from healthy controls and stable from progressive MCI (Casadio et al., 2025). Reduced activation of olfactory and memory-related networks, including OFC, anterior cingulate, and default mode network, occurs during olfactory tasks in MCI (Casadio et al., 2025). Functional near-infrared spectroscopy (fNIRS) has identified oxygenation differences in OFC related to olfactory stimulation and cognitive impairment, showing potential for diagnosing MCI and AD (Kim et al., 2022). Zhang et al. (Zhang et al., 2024) reported altered connectivity in olfactory circuits during odor stimulation in SCD subjects. These findings are consistent with our observed altered activity in regions involved in olfactory and prefrontal processing in SCD, supporting olfactory changes as sensitive markers of cognitive decline. This suggests that olfactory testing may serve as a simple and sensitive screening tool for populations at increased risk for AD; olfactory assessment in SCD may thus help detect early functional abnormalities and support timely clinical intervention. The OFC also plays a key role in cognition and behavior, including learning, memory, and decision-making (Carlen, 2017). Multimodal meta-analyses across the AD spectrum report reduced OFC thickness in AD patients (Tang et al., 2024). MCI patients show altered OFC sulcogyral patterns, possibly representing neurodevelopmental risk markers (Wang et al., 2020). These structural and functional abnormalities in OFC may underlie clinical symptoms such as cognitive decline, olfactory dysfunction, and morphological changes.

Functional connectivity analysis showed that FC of the left ORBmid reduced in the right anterior OFC, while increased in the right inferior temporal gyrus. The left ORBmid is an important limbic structure involved in emotional processing, reward, and executive function (Rudebeck and Rich, 2018). Altered FC of bilateral OFC and piriform cortex in amnestic MCI may underlie olfactory recognition deficits, with increased FC between right OFC and right middle frontal gyrus potentially serving a compensatory role (Tu et al., 2023). These results suggest olfactory network dysfunction in SCD. The right inferior temporal gyrus, involved in higher visual processing and memory, may represent a compensatory activation after hippocampal atrophy in elderly individuals (Belleville et al., 2021), with hippocampal connectivity predicting associative memory (Adnan et al., 2016). Our previously proposed auto-weighted centralized multi-task learning diagnostic model incorporated structural and functional data from hippocampus, middle frontal gyrus, amygdala, inferior temporal gyrus, and medial OFC to identify MCI and SCD (Lei et al., 2021). The observed enhanced cross-hemispheric fronto-temporal connectivity may reflect neural plasticity compensation, supporting maintenance of complex cognitive tasks in early SCD. Notably, we observed a matching elevated FC between the left ORBmid and right inferior temporal gyrus, echoing this bilateral compensatory pattern. In the early state of neurodegenerative diseases, the brain undergoes adaptive neuroplasticity to offset early functional loss. Our FC analyses identified reduced coupling between the left ORBmid and the right anterior OFC, alongside compensatory elevated connectivity from left ORBmid to the right inferior temporal gyrus. This cross-hemispheric compensatory mechanism aligns with the dynamic interhemispheric framework reported by Fan et al. (2026), they found that disrupted homotopic synchronization shifts brain networks toward segregation and enables contralateral regional compensation. At the molecular level, splicing dysregulation serves as an upstream pathogenic driver: aberrant transcription and alternative splicing trigger toxic protein aggregation, transcallosal tract damage and impaired homotopic synchronization, sequentially triggering segregation-state cross-hemispheric compensation (Ran et al., 2026). Future multimodal studies combining transcriptomics and dynamic fMRI will validate the causal link between splicing anomalies and orbitofrontal-temporal network remodeling.

Compared with baseline, 20 Hz-taVNS immediately increased regional neural activity in bilateral olfactory cortices, where SCD patients exhibited baseline reductions. The olfactory cortex is critical for olfaction and memory acquisition, relying on neural connections between olfactory cortex and piriform cortex (Kumar et al., 2021). It also connects to the entorhinal cortex–dorsal hippocampus circuit involved in recognition memory (Salimi et al., 2022). Improvement of olfactory cortex activity by 20 Hz-taVNS suggests potential cognitive benefits through local brain activity modulation. However, as only immediate effects were studied, therapeutic efficacy requires further longitudinal clinical trials.

During 20 Hz-taVNS, FC between left ORBmid and right precentral and postcentral gyri decreased. The precentral gyrus is part of the sensorimotor network (SMN), a center for fine motor coordination, with dysfunction linked to learning, memory decline, and slowed behavioral responses. Damage to the precentral gyrus network is associated with verbal working memory impairment (Cai et al., 2017), and short-term verbal memory dysfunction (Sakurai et al., 2018). The postcentral gyrus plays key roles in somatosensory integration, tactile attention, pain processing, and emotion regulation (Kropf et al., 2019). Enhanced FC among bilateral precentral/postcentral gyri and left paracentral lobule has been associated with somatic symptoms in depression and cognitive disorders (Liu et al., 2021). Notably, SCD patients’ olfactory identification scores correlate closely with tau protein deposition in bilateral precentral gyri, middle temporal gyri, and frontal lobes (Risacher et al., 2017). Our findings of upregulated olfactory cortex activity alongside downregulated sensorimotor network FC could be one form of brain adaptation associated with momentary functional coordination; longitudinal investigations are still needed to confirm this speculation.

Also noteworthy, a single 8-min session of acute 20 Hz-taVNS is insufficient to elicit brain functional remodeling and produce meaningful impacts on the anticipated long-term cognitive and behavioral outcomes. Nevertheless, these findings could lay a theoretical foundation for establishing standardized therapeutic regimens. 20 Hz-taVNS could modulate the regional neural activity and FC of olfactory-related brain regions, whereas 1 Hz-taVNS and staVNS failed to induce significant brain functional alterations. These findings suggest that high-frequency taVNS may be more suitable for the treatment of SCD. As aforementioned, olfactory-related brain regions are critically involved in cognitive function, and structural and functional impairments in these regions occur before the progression to dementia. Notably, olfactory dysfunction emerges even prior to overt cognitive deficits in patients with AD. Relative to baseline measurements, acute 20 Hz-taVNS increased activity in underactive olfactory brain areas and improved disturbed functional connectivity. These acute imaging alterations might indicate a temporary trend of functional normalization and possible brain functional reorganization. Therefore, we speculate that appropriate repetitive taVNS treatment could exert substantial modulatory effects on olfactory-related brain regions, with potential implications for cognitive and olfactory function in patients with preclinical AD. Preclinical optogenetic work demonstrates that repeated high-frequency burst LC stimulation triggers cumulative noradrenergic release and stable cortical network reorganization (Grimm et al., 2024). Human chronic tVNS data further supports this cumulative plasticity logic: 2 weeks of daily stimulation yields sustained improvements in autonomic regulation and affective function in aging populations (Bretherton et al., 2019). Analogously, acute fMRI signatures from single-session rTMS predict long-term therapeutic effects across neuromodulation research (Ge et al., 2022), implying our observed acute imaging metrics could act as preliminary predictive markers for SCD longitudinal trials. Still, direct clinical evidence connecting one-off taVNS brain changes to prolonged cognitive benefits in SCD is lacking, warranting further longitudinal investigation.

Previous studies reported brain responses to various taVNS frequencies and sham stimulation. However, we observed no brain functional changes during 1 Hz-taVNS or sham-taVNS. The LC exhibits two distinct firing modes: tonic firing at 0.5–5 Hz, which maintains baseline norepinephrine (NE) tone, and phasic/burst firing at 10–20 Hz, which produces rapid, large-amplitude NE transients that drive synaptic plasticity and memory consolidation (Aston-Jones and Cohen, 2005; Ludwig et al., 2021). Critically, Grimm et al. (2024) demonstrated using optogenetics that 15 Hz burst-like LC stimulation elicited significantly greater hippocampal NE release and pupil dilation than 3 Hz tonic stimulation, even when total pulse counts were matched, and that the two patterns engaged distinct cortical hierarchies. This indicates that 20 Hz-taVNS, by approximating the phasic firing range of the LC, more effectively recruits the LC–NE pathway than 1 Hz stimulation. Indeed, 1 Hz-taVNS, although capable of modulating inhibitory pain pathways via the descending pain modulation system (Cao et al., 2021), is insufficient to generate the phasic NE bursts required for robust cognitive enhancement. Sham stimulation fails to activate the vagal afferent pathway entirely.

## 5 Limitation

The present study has several limitations: (1) The sample size of this study was relatively small, and it employed a cross-sectional design. Future research should include larger samples and longitudinal designs to improve the reliability of the findings. (2) The use of ReHo and FC analyses can only partially reflect the brain functional characteristics of SCD patients. Future studies should incorporate multimodal imaging techniques to more comprehensively reveal the underlying neural mechanisms. Lian et al. (2026) demonstrated that integrating multimodal neuroimaging (structural, functional, and connectivity) with plasma proteomics via MCCAR+jICA uncover a brain–body pathway linking depression and metabolic dysfunction, revealing disease mechanisms inaccessible to single-modality analysis. Similarly, in oncology, multimodal radiomics fusion significantly refines prognostic stratification over single-modality approaches (Yang et al., 2026). Applying such sophisticated fusion techniques to SCD and early AD—integrating neuroimaging with blood-based biomarkers, holds considerable promise for identifying complex brain–body pathways and improving early diagnostic precision. (3) This study only examined the immediate modulatory effects of taVNS, the sustained efficacy of 20 Hz-taVNS requires further verification. (4) This study identified abnormal activity in the olfaction-related brain regions of SCD patients, which could be modulated by taVNS; however, olfactory function tests were not conducted. Considering the important role of olfaction in AD pathology, future research should include olfactory assessments to better understand the mechanisms of AD.

## 6 Conclusion

SCD patients showed decreased spontaneous neural activity and abnormal FC in the olfaction-related brain regions, which may serve as a central mechanism contributing to cognitive impairment in SCD. Intervention with 20 Hz-taVNS was observed to boost local brain activity and modulate sensorimotor network functional connectivity, potentially inducing transient adjustments in brain function. This approach holds preliminary potential for SCD intervention, yet rigorous longitudinal clinical trials are needed to confirm its actual clinical benefits.

## Data Availability

The raw data supporting the conclusions of this article will be made available by the authors, without undue reservation.

## References

[ref1] Adnan A. Barnett A. Moayedi M. McCormick C. Cohn M. McAndrews M. P. (2016). Distinct hippocampal functional networks revealed by tractography-based parcellation. Brain Struct. Funct. 221, 2999–3012. doi: 10.1007/s00429-015-1084-x, 26206251

[ref2] Aston-Jones G. Cohen J. D. (2005). An integrative theory of locus coeruleus-norepinephrine function: adaptive gain and optimal performance. Annu. Rev. Neurosci. 28, 403–450. doi: 10.1146/annurev.neuro.28.061604.135709 16022602

[ref3] Belleville S. Mellah S. Cloutier S. Dang-Vu T. T. Duchesne S. Maltezos S. . (2021). Neural correlates of resilience to the effects of hippocampal atrophy on memory. Neuroimage Clin. 29:102526. doi: 10.1016/j.nicl.2020.102526 33360019 PMC7770959

[ref4] Bretherton B. Atkinson L. Murray A. Clancy J. Deuchars S. Deuchars J. (2019). Effects of transcutaneous vagus nerve stimulation in individuals aged 55 years or above: potential benefits of daily stimulation. Aging (Albany NY) 11, 4836–4857. doi: 10.18632/aging.102074, 31358702 PMC6682519

[ref5] Broncel A. Bocian R. Kłos-Wojtczak P. Kulbat-Warycha K. Konopacki J. (2020). Vagal nerve stimulation as a promising tool in the improvement of cognitive disorders. Brain Res. Bull. 155, 37–47. doi: 10.1016/j.brainresbull.2019.11.011, 31790720

[ref6] Buckner R. L. Krienen F. M. Yeo B. T. (2013). Opportunities and limitations of intrinsic functional connectivity MRI. Nat. Neurosci. 16, 832–837. doi: 10.1038/nn.3423, 23799476

[ref7] Cai S. Chong T. Peng Y. Shen W. Li J. von Deneen K. M. . (2017). Altered functional brain networks in amnestic mild cognitive impairment: a resting-state fMRI study. Brain Imaging Behav. 11, 619–631. doi: 10.1007/s11682-016-9539-0, 26972578

[ref8] Cao J. Zhang Y. Li H. Yan Z. Liu X. Hou X. . (2021). Different modulation effects of 1 Hz and 20 Hz transcutaneous auricular vagus nerve stimulation on the functional connectivity of the periaqueductal gray in patients with migraine. J. Transl. Med. 19:354. doi: 10.1186/s12967-021-03024-9, 34404427 PMC8371886

[ref9] Carlen M. (2017). What constitutes the prefrontal cortex. Science 358, 478–482. doi: 10.1126/science.aan8868, 29074767

[ref10] Casadio C. Ballotta D. Ricci F. Zanelli V. Carpentiero O. Corni M. G. . (2025). Olfactory testing and gray matter volume: a combined approach to predict the conversion to Alzheimer. Brain Sci. 15:310. doi: 10.3390/brainsci15030310, 40149831 PMC11940542

[ref11] Dai M. Guo Z. Xia H. Zhu H. Li J. Hou H. . (2024). Predicting the efficacy of donepezil intervention in Alzheimer's disease patients using regional homogeneity in the inferior orbitofrontal cortex. Aging Clin. Exp. Res. 36:94. doi: 10.1007/s40520-023-02691-6, 38630202 PMC11024046

[ref12] Dan X. Wechter N. Gray S. Mohanty J. G. Croteau D. L. Bohr V. A. (2021). Olfactory dysfunction in aging and neurodegenerative diseases. Ageing Res. Rev. 70:101416. doi: 10.1016/j.arr.2021.101416, 34325072 PMC8373788

[ref13] Devanand D. P. Tabert M. H. Cuasay K. Manly J. J. Schupf N. Brickman A. M. . (2010). Olfactory identification deficits and MCI in a multi-ethnic elderly community sample. Neurobiol. Aging 31, 1593–1600. doi: 10.1016/j.neurobiolaging.2008.09.008, 18963256 PMC2947189

[ref14] Fan H. Wang H. Lian Z. Yu Q. Wu X. Kuang N. . (2026). Dynamic interactions between hemispheres reveal a compensatory pathway for motor recovery in moderate-to-severe subcortical stroke. J. Stroke 28, 97–114. doi: 10.5853/jos.2025.01725, 41478717 PMC12883883

[ref15] Ge R. Humaira A. Gregory E. Alamian G. MacMillan E. L. Barlow L. . (2022). Predictive value of acute neuroplastic response to rTMS in treatment outcome in depression: a concurrent TMS-fMRI trial. Am. J. Psychiatry 179, 500–508. doi: 10.1176/appi.ajp.21050541, 35582784

[ref16] Grimm C. Duss S. N. Privitera M. Munn B. R. Karalis N. Frässle S. . (2024). Tonic and burst-like locus coeruleus stimulation distinctly shift network activity across the cortical hierarchy. Nat. Neurosci. 27, 2167–2177. doi: 10.1038/s41593-024-01755-8, 39284964 PMC11537968

[ref17] Jack C. R. Jr. Andrews J. S. Beach T. G. Buracchio T. Dunn B. Graf A. . (2024). Revised criteria for diagnosis and staging of Alzheimer's disease: Alzheimer's Association workgroup. Alzheimers Dement. 20, 5143–5169. doi: 10.1002/alz.13859, 38934362 PMC11350039

[ref18] Jessen F. Amariglio R. E. van Boxtel M. Breteler M. Ceccaldi M. Chetelat G. . (2014). A conceptual framework for research on subjective cognitive decline in preclinical Alzheimer's disease. Alzheimers Dement. 10, 844–852. doi: 10.1016/j.jalz.2014.01.001, 24798886 PMC4317324

[ref19] Jobin B. Boller B. Frasnelli J. (2023). Smaller grey matter volume in the central olfactory system in mild cognitive impairment. Exp. Gerontol. 183:112325. doi: 10.1016/j.exger.2023.112325 37952649

[ref20] Kim A. Y. Marduy A. de Melo P. S. Gianlorenco A. C. Kim C. K. Choi H. . (2022). Safety of transcutaneous auricular vagus nerve stimulation (taVNS): a systematic review and meta-analysis. Sci. Rep. 12:22055. doi: 10.1038/s41598-022-25864-1, 36543841 PMC9772204

[ref21] Kim J. Yon D. K. Choi K. Y. Lee J. J. Kim N. Lee K. H. . (2022). Novel diagnostic tools for identifying cognitive impairment using olfactory-stimulated functional near-infrared spectroscopy: patient-level, single-group, diagnostic trial. Alzheimer's Res. Ther. 14:39. doi: 10.1186/s13195-022-00978-w, 35260170 PMC8905807

[ref22] Kropf E. Syan S. K. Minuzzi L. Frey B. N. (2019). From anatomy to function: the role of the somatosensory cortex in emotional regulation. Braz. J. Psychiatry 41, 261–269. doi: 10.1590/1516-4446-2018-0183, 30540029 PMC6794131

[ref23] Kumar A. Barkai E. Schiller J. (2021). Plasticity of olfactory bulb inputs mediated by dendritic NMDA-spikes in rodent piriform cortex. eLife 10:e70383. doi: 10.7554/eLife.70383, 34698637 PMC8575458

[ref24] Lei B. Cheng N. Frangi A. F. Wei Y. Yu B. Liang L. . (2021). Auto-weighted centralised multi-task learning via integrating functional and structural connectivity for subjective cognitive decline diagnosis. Med. Image Anal. 74:102248. doi: 10.1016/j.media.2021.102248, 34597938

[ref25] Lian Z. Liu Z. Fan H. Wang J. Zhang K. Liu Y. . (2026). Multimodal fusion of brain imaging and proteomics reveals a brain-body pathway linking depression and metabolic dysfunction. Psychol. Med. 56:e144. doi: 10.1017/S003329172610436X, 42124391 PMC13200150

[ref26] Lian T. H. Zhu W. L. Li S. W. Liu Y. O. Guo P. Zuo L. J. . (2019). Clinical, structural, and neuropathological features of olfactory dysfunction in patients with Alzheimer's disease. J. Alzheimer's Dis 70, 413–423. doi: 10.3233/JAD-181217, 31177212

[ref27] Liu P. Tu H. Zhang A. Yang C. Liu Z. Lei L. . (2021). Brain functional alterations in MDD patients with somatic symptoms: a resting-state fMRI study. J. Affect. Disord. 295, 788–796. doi: 10.1016/j.jad.2021.08.143, 34517253

[ref28] Ludwig M. Wienke C. Betts M. J. Zaehle T. Hämmerer D. (2021). Current challenges in reliably targeting the noradrenergic locus coeruleus using transcutaneous auricular vagus nerve stimulation (taVNS). Auton. Neurosci. 236:102900. doi: 10.1016/j.autneu.2021.102900, 34781120

[ref29] Murphy A. J. O'Neal A. G. Cohen R. A. Lamb D. G. Porges E. C. Bottari S. A. . (2023). The effects of transcutaneous vagus nerve stimulation on functional connectivity within semantic and hippocampal networks in mild cognitive impairment. Neurotherapeutics 20, 419–430. doi: 10.1007/s13311-022-01318-4, 36477709 PMC10121945

[ref31] Pacyna R. R. Han S. D. Wroblewski K. E. McClintock M. K. Pinto J. M. (2023). Rapid olfactory decline during aging predicts dementia and GMV loss in AD brain regions. Alzheimers Dement. 19, 1479–1490. doi: 10.1002/alz.12717, 35899859

[ref32] Ran X. Wang M. Huang J. Kuang N. Tian P. Wu J. . (2026). Mechanistic research and therapeutic prospects of alternative splicing in neurodegenerative diseases. Ageing Res. Rev. 118:103133. doi: 10.1016/j.arr.2026.103133, 41962593

[ref33] Risacher S. L. Tallman E. F. West J. D. Yoder K. K. Hutchins G. D. Fletcher J. W. . (2017). Olfactory identification in subjective cognitive decline and mild cognitive impairment: association with tau but not amyloid positron emission tomography. Alzheimers Dement. (Amst.) 9, 57–66. doi: 10.1016/j.dadm.2017.09.001, 29159268 PMC5675709

[ref34] Rudebeck P. H. Rich E. L. (2018). Orbitofrontal cortex. Curr. Biol. 28, R1083–R1088. doi: 10.1016/j.cub.2018.07.018, 30253144 PMC9253859

[ref35] Sakurai Y. Furukawa E. Kurihara M. Sugimoto I. (2018). Frontal phonological agraphia and acalculia with impaired verbal short-term memory due to left inferior precentral gyrus lesion. Case Rep. Neurol. 10, 72–82. doi: 10.1159/000487849, 29681826 PMC5903121

[ref36] Salimi M. Tabasi F. Abdolsamadi M. Dehghan S. Dehdar K. Nazari M. . (2022). Disrupted connectivity in the olfactory bulb-entorhinal cortex-dorsal hippocampus circuit is associated with recognition memory deficit in Alzheimer's disease model. Sci. Rep. 12:4394. doi: 10.1038/s41598-022-08528-y, 35292712 PMC8924156

[ref37] Tang X. Guo Z. Chen G. Sun S. Xiao S. Chen P. . (2024). A multimodal meta-analytical evidence of functional and structural brain abnormalities across Alzheimer's disease spectrum. Ageing Res. Rev. 95:102240. doi: 10.1016/j.arr.2024.102240, 38395200

[ref38] Tu L. Wang Z. Lv X. Xie T. Fan Z. Zhang M. . (2023). Characteristics of odor identification and hedonics and their association with piriform cortex-based resting-state functional connectivity in amnestic mild cognitive impairment. J. Alzheimer's Dis 94, 247–258. doi: 10.3233/JAD-221163, 37212099

[ref39] Wang X. Huang W. Su L. Xing Y. Jessen F. Sun Y. . (2020). Neuroimaging advances regarding subjective cognitive decline in preclinical Alzheimer's disease. Mol. Neurodegener. 15:55. doi: 10.1186/s13024-020-00395-3, 32962744 PMC7507636

[ref40] Wang L. Zhang J. Guo C. He J. Zhang S. Wang Y. . (2022). The efficacy and safety of transcutaneous auricular vagus nerve stimulation in patients with mild cognitive impairment: a double blinded randomized clinical trial. Brain Stimul. 15, 1405–1414. doi: 10.1016/j.brs.2022.09.003, 36150665

[ref41] Wang Z. Zhang X. Liu R. Wang Y. Qing Z. Lu J. . (2020). Altered sulcogyral patterns of orbitofrontal cortex in patients with mild cognitive impairment. Psychiatry Res. Neuroimaging 302:111108. doi: 10.1016/j.pscychresns.2020.111108, 32464534

[ref30] World Health Organization. (2024). World Health Statistics 2024- Monitoring Health for the SDGs. Sustainable Development Goals. Geneva: World Health Organization.

[ref42] Yakunina N. Kim S. S. Nam E. C. (2017). Optimization of transcutaneous vagus nerve stimulation using functional MRI. Neuromodulation 20, 290–300. doi: 10.1111/ner.12541, 27898202

[ref43] Yang J. Gohel S. Vachha B. (2020). Current methods and new directions in resting state fMRI. Clin. Imaging 65, 47–53. doi: 10.1016/j.clinimag.2020.04.004, 32353718 PMC7365764

[ref44] Yang Z. Su S. Tan Y. Hu P. Luo X. Kuang N. . (2026). From single-modality to multimodal fusion: research progress of radiomics in prognostic assessment and risk stratification of hepatocellular carcinoma. Cancer Adv. 9:e26007. doi: 10.53388/2026926007

[ref45] Zhang X. Zhu Y. Lu J. Chen Q. Chen F. Long C. . (2024). Altered functional connectivity of primary olfactory cortex-hippocampus-frontal cortex in subjective cognitive decline during odor stimulation. Hum. Brain Mapp. 45:e26814. doi: 10.1002/hbm.26814, 39163575 PMC11335137

